# Therapeutic strategies and outcomes in the management of ampullary tumours: meta-analysis

**DOI:** 10.1093/bjsopen/zrag115

**Published:** 2026-08-26

**Authors:** Anouk J de Wilde, Jan Faes, Karlijn E P E Hermans, Marc G Besselink, Jeska A Fritzsche, Matthias van Haele, Jan-Werner Poley, Rogier P Voermans, Judith De Vos-Geelen, Steven W M Olde Damink, Stefan A W Bouwense

**Affiliations:** Department of Surgery and School of Nutrition and Translational Research in Metabolism (NUTRIM), Maastricht University, Maastricht, the Netherlands; Department of Surgery, Maastricht University Medical Centre+, Maastricht, the Netherlands; Department of Surgery, Maastricht University Medical Centre+, Maastricht, the Netherlands; Department of Internal Medicine, Division of Medical Oncology, GROW—Research Institute for Oncology & Reproduction, Maastricht University Medical Centre+, Maastricht, the Netherlands; Amsterdam UMC, location University of Amsterdam, Department of Surgery, Amsterdam, the Netherlands; Cancer Centre Amsterdam, Amsterdam, the Netherlands; Cancer Centre Amsterdam, Amsterdam, the Netherlands; Department of Gastroenterology and Hepatology, Amsterdam Gastroenterology Endocrinology Metabolism, Amsterdam UMC, University of Amsterdam, Amsterdam, the Netherlands; Department of Pathology, Maastricht University Medical Centre+, Maastricht, the Netherlands; Department of Gastroenterology, Maastricht University Medical Centre+, Maastricht, the Netherlands; Cancer Centre Amsterdam, Amsterdam, the Netherlands; Department of Gastroenterology and Hepatology, Amsterdam Gastroenterology Endocrinology Metabolism, Amsterdam UMC, University of Amsterdam, Amsterdam, the Netherlands; Department of Internal Medicine, Division of Medical Oncology, GROW—Research Institute for Oncology & Reproduction, Maastricht University Medical Centre+, Maastricht, the Netherlands; Department of Surgery and School of Nutrition and Translational Research in Metabolism (NUTRIM), Maastricht University, Maastricht, the Netherlands; Department of Surgery, Maastricht University Medical Centre+, Maastricht, the Netherlands; Department of General, Visceral, Vascular and Transplantation Surgery, University Hospital Essen, Essen, Germany; Department of Surgery and School of Nutrition and Translational Research in Metabolism (NUTRIM), Maastricht University, Maastricht, the Netherlands; Department of Surgery, Maastricht University Medical Centre+, Maastricht, the Netherlands

**Keywords:** endoscopic resection, transduodenal ampullectomy, pancreatoduodenectomy, complete resection, recurrence, complications

## Abstract

**Background:**

Ampullary tumours are rare but present important clinical challenges. This meta-analysis aims to evaluate the outcomes of endoscopic resection (ER), transduodenal ampullectomy (TDA) and pancreatoduodenectomy (PD) for both benign and malignant ampullary tumours.

**Methods:**

A comprehensive search was performed up to 15 December 2025. Studies were included if they reported short-term outcomes including complete resection rate, recurrence, or complications of ampullary tumours after ER, TDA, or PD. Statistical analyses were performed using both random-effects and fixed-effects models to calculate pooled means. Publication bias was evaluated using funnel plots and Egger’s test, and risk of bias was assessed using ROBINS-I V2.

**Results:**

A total of 80 studies were included. For complete resection rate, ER showed high heterogeneity (29%–100%), TDA showed a pooled rate of 90% for benign and early-stage tumours and 66% for malignant tumours, whereas PD showed 99% and 97%, respectively. ER and TDA showed both a pooled recurrence rate of 12% for benign and early-stage tumours, whereas PD showed 6%. For recurrence of malignant tumours, TDA showed high heterogeneity (48%–90%) whereas PD had a pooled rate of 16%. For complications, ER and PD exhibited high heterogeneity (9%–95% and 27%–98%, respectively), whereas TDA had a pooled rate of 32%.

**Conclusion:**

PD provides the best outcomes in terms of complete resection and recurrence, despite higher morbidity. ER is a viable option for benign tumours, offering lower morbidity and manageable recurrence rates. Further prospective research is needed to optimize preoperative diagnostics to distinguish benign tumours from malignant tumours accurately to select the appropriate treatment.

## Teaser

This systematic review compares the outcomes of endoscopic resection, transduodenal ampullectomy, and pancreatoduodenectomy for benign, early-stage, and malignant ampullary tumours. Pancreatoduodenectomy provides the most reliable oncological outcomes with the lowest recurrence rates, whereas endoscopic resection is a suitable option for benign lesions with low morbidity rates. Improved preoperative diagnostics are needed to guide optimal treatment selection.

## Introduction

Tumours of the ampulla of Vater are rare, and account for 7–10% of all periampullary tumours^1,2^. Ampullary tumours can be classified into benign and malignant, with adenomas being the most common benign diagnosis and adenocarcinomas presenting the most common malignant form. Ampullary adenomas are considered potential premalignant lesions, with some having a risk of progression to carcinoma^3,4^.

For ampullary adenomas, endoscopic resection (ER) is the primary treatment if the tumour is considered technically resectable by endoscopy^5,6^. Factors such as intraductal extension and extent of (circumferential) growth may limit the feasibility of ER^1^. In these cases, transduodenal ampullectomy (TDA) or pancreatoduodenectomy (PD) may be considered as alternative treatment options^7^. For ampullary adenocarcinomas, an oncological resection is indicated and PD is the primary treatment. The viability of local treatment options such as ER or TDA for early-stage adenocarcinomas (Tis and T1) remains unclear in the literature. Some studies^8,9^ show that these approaches yield acceptable results, with lower morbidity and mortality compared with PD. In contrast, other studies indicate that the oncological outcomes of local treatments are inferior to PD, primarily due to the absence of lymph node dissection and inaccuracy of preoperative diagnostic methods in staging ampullary tumours. Additionally, T1 tumours have a high likelihood of lymph node involvement, suggesting that PD remains the only curative option^10,11^.

Beyond the indication, the outcomes of these treatments should also be considered, as they can vary substantially. ER is a less invasive option, associated with relatively low morbidity and mortality rates^7,12^, though recurrence has been reported in up to approximately 30% of patients^13–15^. TDA carries higher morbidity and mortality compared with ER, with variable recurrence rates reported, sometimes reaching up to 80%^16^. PD, the most radical approach, is associated with the highest morbidity and mortality rates^17,18^.

Despite increasing clinical experience and advances in endoscopic and surgical techniques, conclusive evidence regarding the comparative effectiveness and safety of ER, TDA, and PD remains limited, partly due to the lack of prospective studies. A better understanding of the outcomes of these treatment options is essential to improve clinical decision-making and outcomes. Therefore, the objective of this meta-analysis is to evaluate the rates of complete resection, recurrence, and complications in ER, TDA, and PD for ampullary tumours.

## Methods

This meta-analysis was conducted according to the Cochrane Handbook and the PRISMA guidelines and was registered in the PROSPERO database (CRD420251044805)^19–21^.

### Search strategy

A systematic search was conducted in PubMed, Embase, CINAHL, and the Cochrane Library databases to identify relevant studies evaluating therapeutic strategies and outcomes in the management of ampullary tumours. The studies included randomized clinical trials (RCTs) and (comparative) observational studies. No specific minimum volume of patients per study was applied to the search. The search was conducted from database inception up to 15 December 2025 using the following search terms: ‘Ampulla of Vater’, ‘Neoplasms’, ‘Common Bile Duct Neoplasm’, ‘Pancreaticoduodenectomy’, ‘Transduodenal ampullectomy’, ‘Endoscopic resection’, ‘complete resection’, ‘recurrence’, complication’. Synonyms of these terms were also included in the search. The complete search is described in the *Supplementary Appendixes a–d*. There were no restrictions on language or publication dates. Duplicate references were removed, and all results were uploaded into Rayyan, a web-based application used for screening eligible studies for systematic reviews^22^.

### Study selection

All articles were independently screened by two reviewers (A.dW. and J.F.) based on the prespecified inclusion and exclusion criteria using title and abstract. Studies were included if they met the following inclusion criteria: patients aged 18 years or older, diagnosed with ampullary tumours (both benign and malignant), who had undergone resection procedures for their ampullary tumour such as ER, TDA, or PD, with outcomes including complete resection rate, recurrence, or complication rate being reported. Studies were excluded if they involved patients with other types of gastrointestinal tumours (that is, gastrointestinal stromacell tumours, neuroendocrine neoplasms), were reviews, letters, book chapters, or case reports, or included fewer than ten patients. After screening the abstracts, the two reviewers independently assessed the full text of potentially relevant articles to complete the final selection. Any disagreements were resolved through discussion, and when consensus could not be reached, an arbitrator (S.B.) made the final decision.

### Data extraction and data collection

Two reviewers (A.dW. and J.F.) independently screened all articles, extracted data, and assessed the risk of bias. A standardized data extraction form was used to collect relevant information, including the first author, year of publication, study design (for example, prospective or retrospective cohort study or cross-sectional study), total number of patients, patient demographics, preoperative diagnosis, type of intervention, complete resection rate (defined as R0 resection for TDA and PD and, for ER, R0 resection or no residual tumour during first follow-up endoscopy in case of Rx (resection margin could not be assessed)), residual disease (defined as residual tumour during first follow-up endoscopy), recurrence (locoregional), postoperative complication rate, in-hospital mortality, and postoperative diagnosis. Because definitions of outcome measures varied across studies, particularly for complete resection and postoperative complications, outcomes were extracted as reported in the original studies. For this review, tumours were categorized as benign tumours (adenomas with low- and high-grade dysplasia), early-stage tumours (Tis and T1 adenocarcinomas), or malignant (> T1 adenocarcinomas) based on final histopathological diagnosis. Many studies report results after endoscopic or local surgical resection, including adenomas, and Tis and T1 tumours without distinction into subgroups. Therefore, benign tumours and early-stage tumours were combined for analysis.

### Risk of bias

The ROBINS-I V2 tool was used to assess the risk of bias in the included studies^23^. This tool evaluated potential bias in seven domains: confounding, participant selection, classification of interventions, deviations from intended interventions, missing data, outcome measurement, and selection of the reported outcome. Each domain was rated for risk of bias, and the overall rating was categorized as low, moderate, serious, or critical, in accordance with the guidelines.

### Statistical analysis

Statistical analyses were conducted using R Studio version 4.5.0. Meta-analyses were performed for the primary outcomes: complete resection, recurrence, and complications. Subgroup analyses for complete resection and recurrence in TDA and PD were conducted using two groups: benign and early-stage tumours (Tis and T1), and malignant tumours. Both random-effects and fixed-effects models were used, depending on the heterogeneity of the studies. Fixed-effects models were applied when heterogeneity was low or moderate (*I*^2^ < 50%), whereas random-effects models were used for outcomes with high heterogeneity (*I*^2^ > 50%). Heterogeneity was assessed using the *I*^2^ statistic and visual inspection of forest plots. For outcomes with high heterogeneity (*I*^2^ > 60%), sensitivity analyses were performed. In cases where heterogeneity remained high despite excluding studies, individual study results were presented without pooling. Publication bias was assessed using Egger’s test and funnel plots. All *P*-values were two-sided, with *P* < 0.05 considered statistically significant.

## Results

### Study characteristics

**Fig. 1:**
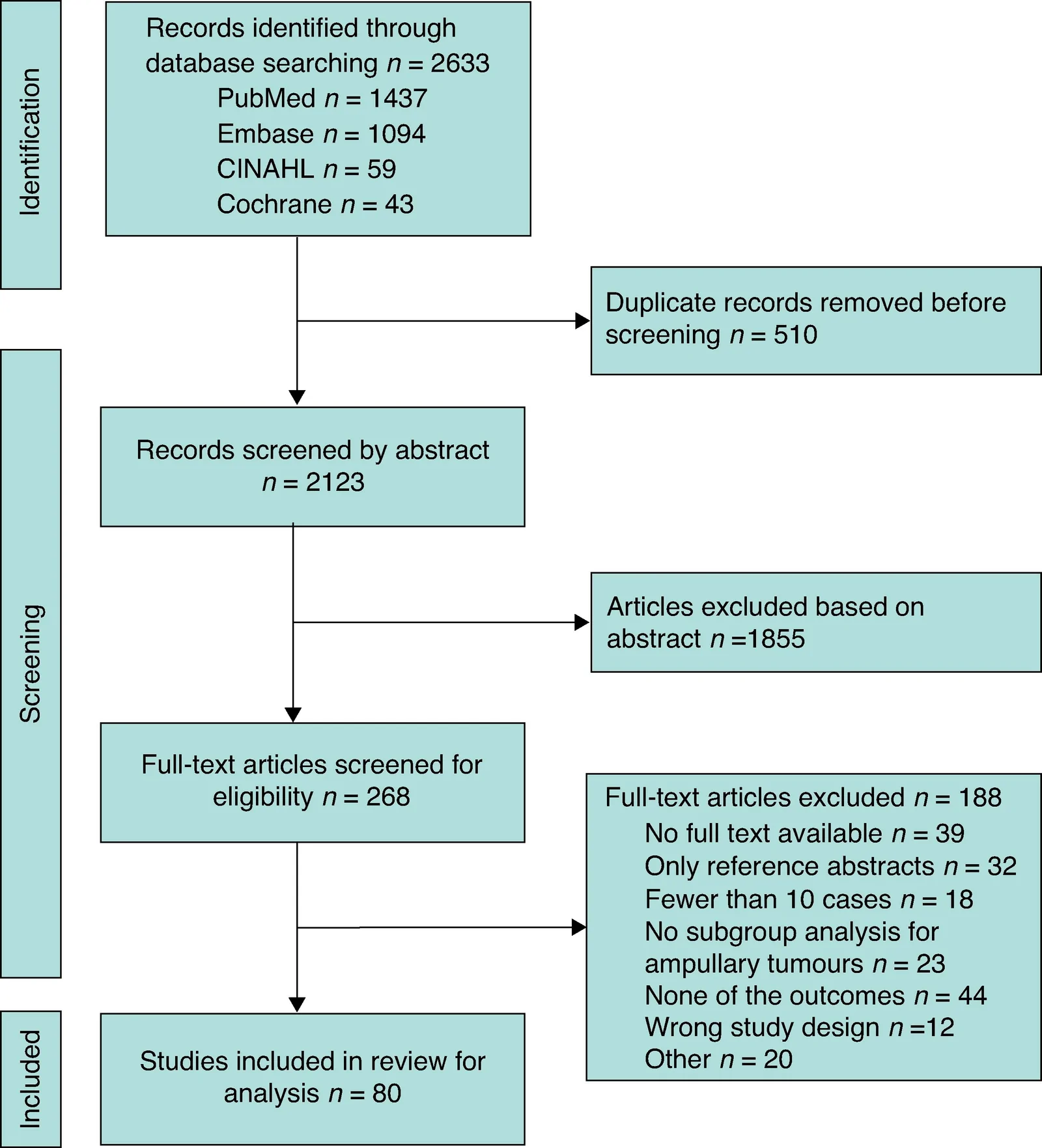
PRISMA flowchart showing the search strategy and study selection process

The systematic search yielded a total of 2633 articles. After screening the abstracts, 268 articles were identified for full-text review, of which 80 studies were ultimately included for analyses (*Fig. 1*)^8,9,13–15,17,24–97^. Study characteristics are presented in *Table 1*. Of the studies included, 77 were retrospective cohort studies, and only 3 were prospective cohort studies. A total of 7904 patients were included. Among the studies, 37 included only benign tumours, 30 included both benign and early-stage tumours (Tis and T1), 1 included only early-stage tumours, 2 included both benign and malignant tumours, and 10 included only malignant tumours. Multiple interventions were reported in 17 studies, but none directly compared interventions. Among the included studies, 49 focused on ER, 4 on both ER and PD, 6 on ER and TDA, 8 on PD, 6 on TDA, and 7 on both TDA and PD. Consequently, 59 studies reported on ER, 19 on PD, and 19 on TDA. Outcomes reported in the studies are summarized in *Table 2* and are discussed below.

**Table 1:** Characteristics of the included studies

Study	Year	Country	Patients (*n*)	Study design	Tumour diagnosis	Intervention
Abe *et al*.^24^	2022	Japan	74	Retrospective, single centre	Benign and early-stage	ER + PD
Ahn *et al*.^25^	2013	Korea	43	Retrospective, single centre	Benign	ER
Alali *et al*.^26^	2020	Canada	103	Retrospective, single centre	Benign and early-stage	ER
Alvarez-Sanchez *et al.*^27^	2017	France	28	Retrospective, single centre	Early-stage	ER
Attila *et al*.^28^	2018	Turkey	44	Retrospective, multicentre	Benign	ER
Bohnacker *et al*.^29^	2005	Germany	106	Retrospective, single centre	Benign	ER
Boix *et al*.^30^	2009	Spain	21	Retrospective, single centre	Benign	ER
Catalano *et al*.^13^	2004	United States	103	Retrospective, multicentre	Benign	ER
Chapman *et al*.^31^	2018	United States	47	Retrospective, single centre	Malignant	PD
Chavez *et al*.^17^	2017	United States	60	Retrospective, single centre	Malignant	PD
Cheng *et al*.^14^	2004	United States	55	Retrospective, single centre	Benign	ER
Choi *et al*.^32^	2022	South Korea	196	Retrospective, single centre	Benign	ER
Choi *et al*.^33^	2024	South Korea	106	Retrospective, multicentre	Benign	ER
de Palma *et al*.^34^	2015	Italy	27	Retrospective, single centre	Benign	ER
Dembinski *et al*.^35^	2022	France	93	Prospective, single centre	Malignant	PD
Demetriades *et al*.^36^	2016	Greece	20	Retrospective, single centre	Benign and early-stage	TDA
Dubois *et al*.^37^	2017	Switzerland	30	Retrospective, single centre	Benign and early-stage	ER +TDA
Feng *et al*.^38^	2012	China	71	Retrospective, single centre	Malignant	TDA + PD
Fritzsche *et al*.^39^	2021	Netherlands	259	Retrospective, multicentre	Benign	ER
Gambitta *et al*.^40^	2021	Italy	30	Retrospective, single centre	Benign	ER
Gao *et al*.^41^	2023	China	43	Prospective, single centre	Benign and early-stage	TDA + PD
Ghidrim *et al*.^42^	2009	Moldova	12	Retrospective, single centre	Benign	ER
Gondran *et al*.^43^	2022	France	227	Retrospective, multicentre	Benign and early-stage	ER
Gracient *et al*.^44^	2020	France	51	Retrospective, single centre	Benign and early-stage	ER +TDA
Grobmyer *et al*.^45^	2008	United States	29	Retrospective, single centre	Benign	TDA
Harano *et al*.^46^	2011	Japan	28	Retrospective, single centre	Benign	ER
Hollenbach *et al*.^47^	2025	Germany	632	Retrospective, multicentre	Benign and early-stage	ER +TDA
Hong *et al*.^48^	2018	South Korea	26	Retrospective, single centre	Benign	TDA
Hong *et al*.^49^	2021	Korea	494	Retrospective, multicentre	Benign and early-stage	TDA + PD
Hwang *et al*.^50^	2021	South Korea	70	Retrospective, single centre	Benign and early-stage	ER
Irani *et al*.^51^	2009	United States	102	Retrospective, single centre	Benign	ER
Jeanniard *et al*.^52^	2011	France	42	Retrospective, single centre	Benign and early-stage	ER
Jung *et al*.^53^	2009	South Korea	22	Retrospective, single centre	Benign and early-stage	ER
Jung *et al*.^54^	2021	South Korea	31	Retrospective, single centre	Benign and early-stage	TDA
Kang *et al*.^55^	2017	South Korea	104	Retrospective, multicentre	Benign	ER
Kawashima *et al*.^56^	2021	Japan	253	Retrospective, single centre	Benign	ER
Kim *et al*.^58^	2011	Korea	31	Retrospective, single centre	Benign	ER +TDA
Kim and Choi^57^	2017	South Korea	43	Retrospective, single centre	Benign and malignant	ER +TDA
Klein *et al*.^59^	2018	Australia	125	Retrospective, single centre	Benign	ER
Lai *et al*.^60^	2015	Taiwan	377	Retrospective, single centre	Benign	TDA + PD
Laleman *et al*.^61^	2013	Belgium	91	Retrospective, single centre	Benign and early-stage	ER
Lee *et al*. (Junghwan)^63^	2024	South Korea	129	Retrospective, single centre	Benign and early-stage	ER
Lee *et al*. (Jonghyun)^64^	2024	South Korea	37	Retrospective, single centre	Benign and early-stage	ER
Lee *et al*.^65^	2021	Australia	53	Retrospective, single centre	Benign and early-stage	ER
Lee *et al*.^62^	2016	South Korea	137	Retrospective, single centre	Benign	TDA + PD
Li *et al*.^66^	2019	China	110	Retrospective, single centre	Benign	ER
Lindell *et al*.^67^	2003	Sweden	59	Retrospective, single centre	Malignant	TDA + PD
Lu *et al*.^68^	2020	China	72	Retrospective, single centre	Benign	ER
Lv *et al*.^69^	2022	China	95	Retrospective, single centre	Benign and early-stage	ER +PD
Meneghetti *et al*.^70^	2005	United States	30	Retrospective, multicentre	Benign and early-stage	TDA
Min *et al*.^71^	2022	South Korea	179	Retrospective, single centre	Benign and early-stage	TDA + PD
Miyamoto *et al*.^72^	2023	Japan	70	Retrospective, single centre	Benign	ER
Muro *et al*.^73^	2021	Japan	46	Retrospective, single centre	Benign	ER
Napoleon *et al*.^8^	2014	France	93	Prospective, multicentre	Benign and early-stage	ER
Nappo *et al*.^74^	2020	Italy	36	Retrospective, single centre	Benign and early-stage	TDA
Norton *et al*.^15^	2002	United States	28	Retrospective, single centre	Benign	ER
Odemis *et al*.^75^	2020	Turkey	29	Retrospective, single centre	Benign	ER
Onkendi *et al*.^76^	2014	United States	180	Retrospective, single centre	Benign and early-stage	ER +PD
Patel *et al*.^77^	2011	United States	38	Retrospective, single centre	Benign	ER
Perez-Cuadrado-Robles *et al*.^78^	2019	Belgium	73	Retrospective, single centre	Benign	ER
Ridtitid *et al*.^79^	2014	United States	182	Retrospective, single centre	Benign	ER
Russell *et al.*^80^	2023	UK	70	Retrospective, single centre	Malignant	PD
Sahar *et al*.^81^	2020	United States	161	Retrospective, single centre	Benign	ER
Sakai *et al*.^82^	2019	Japan	45	Retrospective, single centre	Benign and early-stage	ER
Sakuma *et al*.^83^	2024	Japan	55	Retrospective, single centre	Malignant	TDA + PD
Saurin *et al*.^84^	2003	France	24	Retrospective, single centre	Benign	ER
Sekine *et al*.^85^	2021	Japan	42	Retrospective, single centre	Benign and early-stage	ER +TDA
Seyfried *et al*.^86^	2022	Germany	85	Retrospective, single centre	Benign and malignant	ER +PD
Shim *et al*.^87^	2014	Korea	39	Retrospective, single centre	Benign	ER
Shyr *et al*.^88^	2024	Taiwan	147	Retrospective, single centre	Malignant	PD
Suzuki *et al*.^89^	2024	Japan	30	Retrospective, multicentre	Benign and early-stage	ER
Tringali *et al*.^90^	2020	Italy	135	Retrospective, single centre	Benign and early-stage	ER
van der Wiel *et al*.^91^	2019	Netherlands	87	Retrospective, single centre	Benign	ER
Wang *et al*.^92^	2020	China	56	Retrospective, single centre	Benign	ER
Will *et al*.^93^	2013	Germany	54	Prospective, multicentre	Benign and early-stage	ER
Xie *et al*.^94^	2024	China	106	Retrospective, single centre	Benign and early-stage	ER
Yamamoto *et al*.^9^	2019	Japan	167	Retrospective, single centre	Benign and early-stage	ER
Yoo *et al*.^95^	2020	South Korea	359	Retrospective, single centre	Malignant	PD
Yoon *et al*.^96^	2005	South Korea	201	Retrospective, single centre	Malignant	PD
Zadorova *et al*.^97^	2001	Czech Republic	16	Retrospective, single centre	Benign	ER

ER, endoscopic resection; PD, pancreatoduodenectomy; TDA, transduodenal ampullectomy.

**Table 2:** Outcomes of the included studies

Study	Year	Intervention	Tumour diagnosis	Patients	Complete resection	Recurrence	Morbidity	Mortality
Abe *et al*.^24^	2022	ER	Benign and early-stage	43	21 (49%)	7 (16%)	25 (58%)	0 (0%)
Ahn *et al*.^25^	2013	ER	Benign and early-stage	43	37 (86%)	2 (5%)	14 (33%)	0 (0%)
Alali *et al*.^26^	2020	ER	Benign and early-stage	103	85 (82.5%)	30 (29.1%)	48 (46.6%)	0 (0%)
Alvarez-Sanchez *et al*.^27^	2017	ER	Benign and early-stage	28	16 (57%)	2 (7%)	NA	NA
Attila *et al*.^28^	2018	ER	Benign and early-stage	44	40 (91%)	7 (16%)	7 (16%)	0 (0%)
Bohnacker *et al*.^29^	2005	ER	Benign and early-stage	106	73 (68.9%)	15 (14.2%)	40 (37.7%)	0 (0%)
Boix *et al*.^30^	2009	ER	Benign and early-stage	21	6 (29%)	1 (5%)	5 (24%)	0 (0%)
Catalano *et al*.^13^	2004	ER	Benign and early-stage	103	NA	10 (9.7%)	10 (9.7%)	1 (1.0%)
Cheng *et al*.^14^	2004	ER	Benign and early-stage	55	37 (67%)	9 (16%)	10 (18%)	0 (0%)
Choi *et al*.^32^	2022	ER	Benign and early-stage	106	84 (79.2%)	24 (22.6%)	26 (24.5%)	0 (0%)
Choi *et al*.^33^	2024	ER	Benign and early-stage	196	184 (93.9%)	27 (13.8%)	89 (45.4%)	0 (0%)
de Palma *et al*.^34^	2015	ER	Benign and early-stage	27	24 (27%)	1 (4%)	5 (19%)	0 (0%)
Dubois *et al*.^37^	2017	ER	Benign and early-stage	11	NA	NA	1 (9%)	0 (0%)
Fritzsche *et al*.^39^	2021	ER	Benign and early-stage	259	NA	24 (9.3%)	50 (19.3%)	1 (0.4%)
Gambitta *et al*.^40^	2021	ER	Benign and early-stage	30	25 (83%)	2 (7%)	7 (23%)	0 (0%)
Ghidirim *et al*.^42^	2009	ER	Benign and early-stage	12	10 (83%)	0 (0%)	3 (25%)	0 (0%)
Gondran *et al*.^43^	2022	ER	Benign and early-stage	227	102 (44.9%)	64 (28.2%)	83 (36.6%)	0 (0%)
Gracient *et al*.^44^	2020	ER	Benign and early-stage	27	19 (70%)	6 (22%)	3 (11%)	0 (0%)
Harano *et al*.^46^	2011	ER	Benign and early-stage	28	NA	3 (11%)	9 (32%)	0 (0%)
Hollenbach *et al*.^47^	2024	ER	Benign and early-stage	569	416 (73.1%)	82 (14.4%)	NA	0 (0%)
Hwang *et al*.^50^	2021	ER	Benign and early-stage	42	24 (57%)	11 (26%)	NA	0 (0%)
Irani *et al*.^51^	2009	ER	Benign and early-stage	102	NA	8 (7.8%)	21 (20.6%)	1 (1.0%)
Jeanniard *et al*.^52^	2011	ER	Benign and early-stage	42	39 (93%)	4 (10%)	10 (24%)	0 (0%)
Jung *et al*.^53^	2009	ER	Benign and early-stage	22	12 (55%)	NA	5 (23%)	0 (0%)
Kang *et al*.^55^	2017	ER	Benign and early-stage	104	93 (89.4%)	7 (6.7%)	42 (40.4%)	2 (1.9%)
Kawashima *et al*.^56^	2021	ER	Benign and early-stage	253	106 (41.9%)	24 (9.5%)	NA	NA
Kim *et al*.^58^	2011	ER	Benign and early-stage	22	16 (73%)	2 (9%)	11 (50%)	0 (0%)
Kim and Choi^57^	2017	ER	Benign and early-stage	10	10 (100%)	1 (10%)	5 (50%)	0 (0%)
Klein *et al*.^59^	2018	ER	Benign and early-stage	125	122 (97.6%)	18 (14.4%)	37 (29.6%)	0 (0%)
Laleman *et al*.^61^	2013	ER	Benign and early-stage	91	84 (92%)	13 (14%)	23 (25%)	0 (0%)
Lee *et al*. (Junghwan)^63^	2024	ER	Benign and early-stage	129	82 (63.6%)	28 (21.7%)	NA	NA
Lee *et al*. (Jonghyun)^64^	2024	ER	Benign and early-stage	37	23 (62%)	14 (7%)	32 (86%)	0 (0%)
Lee *et al*.^65^	2021	ER	Benign and early-stage	53	44 (83%)	7 (13%)	10 (19%)	0 (0%)
Li *et al*.^66^	2019	ER	Benign and early-stage	110	88 (80.0%)	13 (11.8%)	46 (41.8%)	0 (0%)
Lu *et al*.^68^	2020	ER	Benign and early-stage	72	72 (100%)	7 (10%)	9 (12%)	0 (0%)
Lv *et al*.^69^	2022	ER	Benign and early-stage	58	34 (59%)	8 (14%)	15 (26%)	0 (0%)
Miyamoto *et al*.^72^	2023	ER	Benign and early-stage	70	27 (39%)	1 (1%)	23 (33%)	0 (0%)
Muro *et al*.^73^	2021	ER	Benign and early-stage	46	19 (41%)	7 (15%)	29 (63%)	0 (0%)
Napoleon *et al*.^8^	2014	ER	Benign and early-stage	93	85 (91%)	5 (5%)	39 (42%)	1 (1%)
Norton *et al*.^15^	2002	ER	Benign and early-stage	28	14 (50%)	2 (7%)	7 (25%)	0 (0%)
Odemis *et al*.^75^	2020	ER	Benign and early-stage	29	21 (72%)	4 (14%)	6 (21%)	0 (0%)
Onkendi *et al*.^76^	2014	ER	Benign and early-stage	130	72 (55.4%)	44 (33.8%)	38 (29.2%)	0 (0%)
Patel *et al*.^77^	2011	ER	Benign and early-stage	38	33 (87%)	6 (16%)	6 (16%)	0 (0%)
Perez-Cuadrado-Robles *et al*.^78^	2019	ER	Benign and early-stage	73	28 (38%)	NA	15 (21%)	0 (0%)
Ridtitid *et al*.^79^	2014	ER	Benign and early-stage	182	107 (58.8%)	16 (8.8%)	34 (18.7%)	1 (0.5%)
Sahar *et al*.^81^	2020	ER	Benign and early-stage	161	130 (80.7%)	8 (5.0%)	24 (14.9%)	0 (0%)
Sakai *et al*.^82^	2019	ER	Benign and early-stage	45	21 (47%)	4 (9%)	22 (49%)	0 (0%)
Saurin *et al*.^84^	2003	ER	Benign and early-stage	24	16 (67%)	1 (4%)	7 (29%)	0 (0%)
Sekine *et al*.^85^	2021	ER	Benign and early-stage	33	23 (70%)	3 (9%)	7 (21%)	0 (0%)
Seyfried *et al*.^86^	2022	ER	Benign and early-stage	42	NA	3 (7%)	40 (95%)	0 (0%)
Shim *et al*.^87^	2014	ER	Benign and early-stage	39	29 (74%)	5 (13%)	11 (28%)	0 (0%)
Suzuki *et al*.^89^	2023	ER	Benign and early-stage	30	18 (60%)	2 (7%)	17 (57%)	1 (3%)
Tringali *et al*.^90^	2020	ER	Benign and early-stage	135	110 (81.5%)	24 (17.8%)	29 (21.5%)	1 (0.7%)
van der Wiel *et al*.^91^	2019	ER	Benign and early-stage	87	NA	10 (11%)	22 (25%)	0 (0%)
Wang *et al*.^92^	2020	ER	Benign and early-stage	56	52 (93%)	7 (12%)	41 (73%)	0 (0%)
Will *et al*.^93^	2013	ER	Benign and early-stage	54	44 (81%)	10 (19%)	10 (19%)	0 (0%)
Xie *et al*.^94^	2024	ER	Benign and early-stage	61	37 (61%)	8 (13%)	12 (20%)	0 (0%)
Yamamoto *et al*.^9^	2019	ER	Benign and early-stage	167	121 (72.5%)	NA	64 (38.3%)	0 (0%)
Zadorova *et al*.^97^	2001	ER	Benign and early-stage	16	NA	3 (19%)	2 (12%)	0 (0%)
Abe *et al*.^25^	2022	PD	Benign and early-stage	31	31 (100%)	1 (3%)	16 (52%)	0 (0%)
Chapman *et al*.^31^	2018	PD	Malignant	47	47 (100%)	7 (15%)	46 (98%)	0 (0%)
Chavez *et al*.^17^	2017	PD	Malignant	60	55 (92%)	2 (3%)	34 (57%)	3 (5%)
Dembinski *et al*.^35^	2022	PD	Malignant	93	82 (88%)	8 (9%)	83 (89%)	3 (3%)
Feng *et al*.^38^	2012	PD	Malignant	46	43 (93%)	10 (22%)	14 (30%)	3 (7%)
Gao *et al*.^41^	2023	PD	Benign and early-stage	19	19 (100%)	0 (0%)	7 (37%)	0 (0%)
Hong *et al*.^49^	2021	PD	Benign and early-stage	418	417 (99.8%)	23 (5.5%)	NA	NA
Lai *et al*.^60^	2015	PD	Malignant	362	NA	70 (19.3%)	183 (50.6%)	35 (9.7%)
Lee *et al*.^62^	2016	PD	Benign and early-stage	119	NA	4 (3.4%)	51 (42.9%)	1 (0.8%)
Lindell *et al*.^67^	2003	PD	Malignant	49	37 (76%)	11 (22%)	29 (58%)	3 (6%)
Lv *et al*.^69^	2022	PD	Benign and early-stage	37	37 (100%)	0 (0%)	22 (59%)	2 (5%)
Min *et al*.^71^	2022	PD	Benign and early-stage	133	133 (100%)	11 (8.3%)	100 (75.2%)	3 (2.3%)
Onkendi *et al*.^76^	2014	PD	Benign and early-stage	50	NA	NA	29 (58%)	1 (2%)
Russell *et al*.^80^	2023	PD	Malignant	70	53 (76%)	38 (54%)	NA	3 (4%)
Sakuma *et al*.^83^	2024	PD	Malignant	55	54 (98%)	10 (18%)	27 (49%)	0 (0%)
Seyfried *et al*.^86^	2022	PD	Malignant	43	43 (100%)	0 (0%)	27 (63%)	3 (7%)
Shyr *et al*.^88^	2024	PD	Malignant	147	145 (98.6%)	NA	89 (60.5%)	1 (0.7%)
Yoo *et al*.^95^	2020	PD	Malignant	359	354 (98.6%)	NA	97 (27.0%)	0 (0%)
Yoon *et al*.^96^	2005	PD	Malignant	201	192 (95.5%)	NA	68 (33.8%)	2 (1.0%)
Yoon *et al*.^96^	2005	PD	Benign and early-stage	67	NA	4 (6)	NA	1 (1)
Demetriades *et al*.^36^	2016	TDA	Benign and early-stage	20	20 (100%)	3 (15%)	2 (10%)	0 (0%)
Dubois *et al*.^37^	2017	TDA	Benign and early-stage	19	NA	NA	13 (68%)	0 (0%)
Feng *et al*.^38^	2012	TDA	Malignant	25	19 (76%)	12 (48%)	2 (8%)	0 (0%)
Gao *et al*.^41^	2023	TDA	Benign and early-stage	24	24 (100%)	0 (0%)	8 (33%)	0 (0%)
Gracient *et al*.^44^	2020	TDA	Benign and early-stage	24	24 (100%)	0 (0%)	9 (38%)	0 (0%)
Grobmyer *et al*.^45^	2008	TDA	Benign and early-stage	29	NA	3 (10%)	13 (45%)	1 (3%)
Hollenbach *et al*.^47^	2024	TDA	Benign and early-stage	63	57 (90%)	3 (5%)	NA	1 (2)
Hong *et al*.^50^	2021	TDA	Benign and early-stage	68	62 (91%)	11 (16%)	NA	NA
Hong *et al*.^48^	2018	TDA	Benign and early-stage	26	NA	2 (8%)	8 (31%)	0 (0%)
Jung *et al*.^54^	2021	TDA	Benign and early-stage	31	NA	1 (3%)	5 (16%)	0 (0%)
Kim *et al*.^58^	2011	TDA	Benign and early-stage	21	20 (95%)	1 (5%)	5 (24%)	0 (0%)
Kim and Choi^57^	2017	TDA	Benign and early-stage	21	21 (100%)	0 (0%)	5 (24%)	0 (0%)
Lai *et al*.^60^	2015	TDA	Benign and early-stage	15	15 (100%)	2 (13%)	3 (20%)	0 (0%)
Lee *et al*.^62^	2016	TDA	Benign and early-stage	18	NA	2 (11%)	6 (18%)	0 (0%)
Lindell *et al*.^67^	2003	TDA	Malignant	10	5 (50%)	8 (80%)	2 (10%)	0 (0%)
Meneghetti *et al*.^70^	2005	TDA	Benign and early-stage	30	24 (80%)	5 (17%)	7 (30%)	0 (0%)
Min *et al*.^71^	2022	TDA	Benign and early-stage	46	46 (100%)	5 (11%)	17 (46%)	0 (0%)
Nappo *et al*.^74^	2020	TDA	Benign and early-stage	36	32 (89%)	7 (19%)	16 (44%)	0 (0%)
Sekine *et al*.^85^	2021	TDA	Benign and early-stage	9	7 (78%)	2 (22%)	3 (33%)	0 (0%)

Values are *n* (%). ER, endoscopic resection; NA, not applicable; PD, pancreatoduodenectomy; TDA, transduodenal ampullectomy.

### Complete resection

**Fig. 2:**
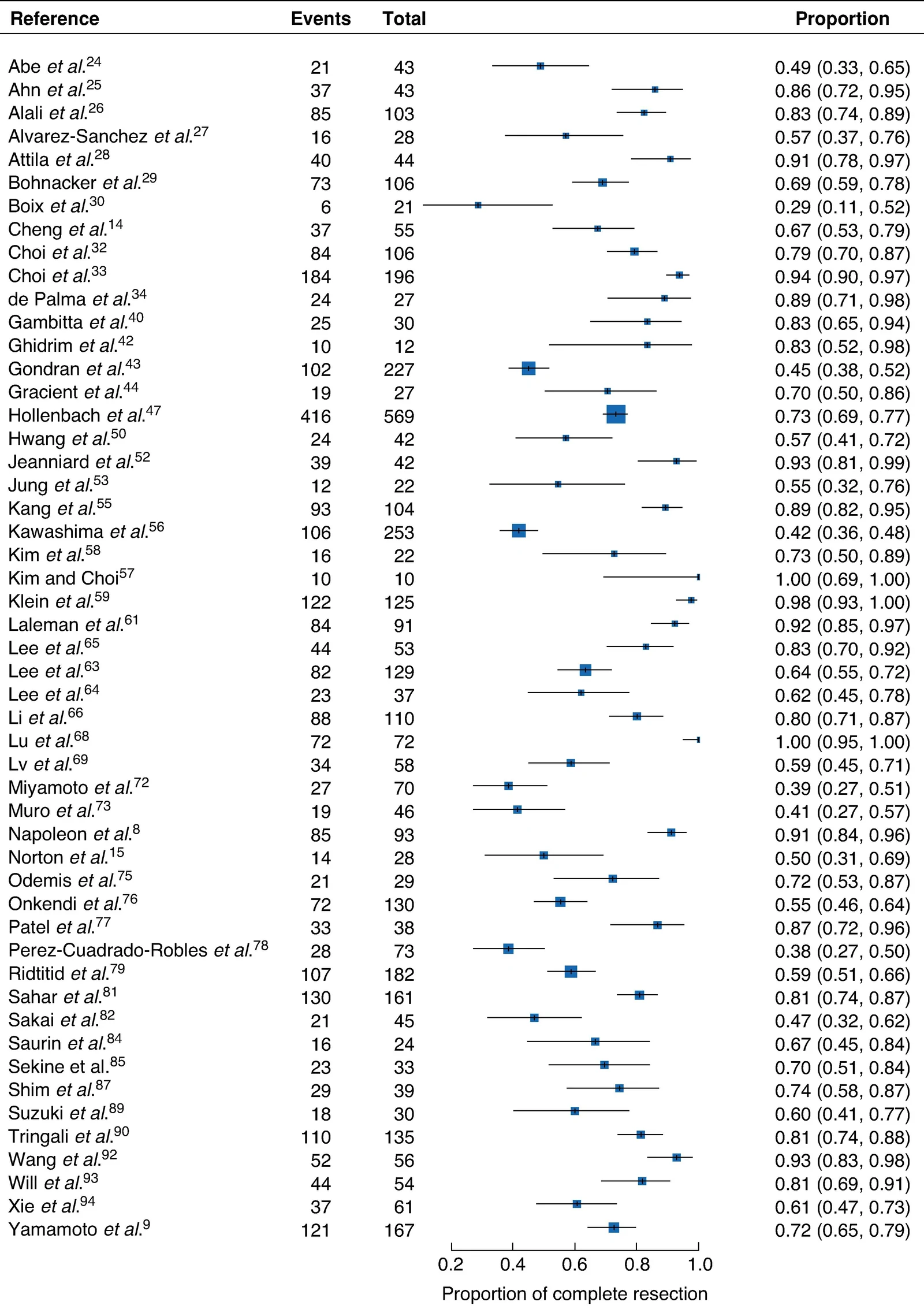
Forest plot reporting complete resection rate in endoscopic resection for benign and early-stage tumours

Data on complete resection in ER were reported in 51 of 59 included studies. A proportion meta-analysis was attempted but showed a high heterogeneity (*I*^2^ = 89.9%; *P* < 0.001). After conducting sensitivity analysis, the heterogeneity remained substantial; consequently, no pooled analysis was performed. The complete resection rates for ER ranged from 29% to 100% (*Fig. 2*).

**Fig. 3:**
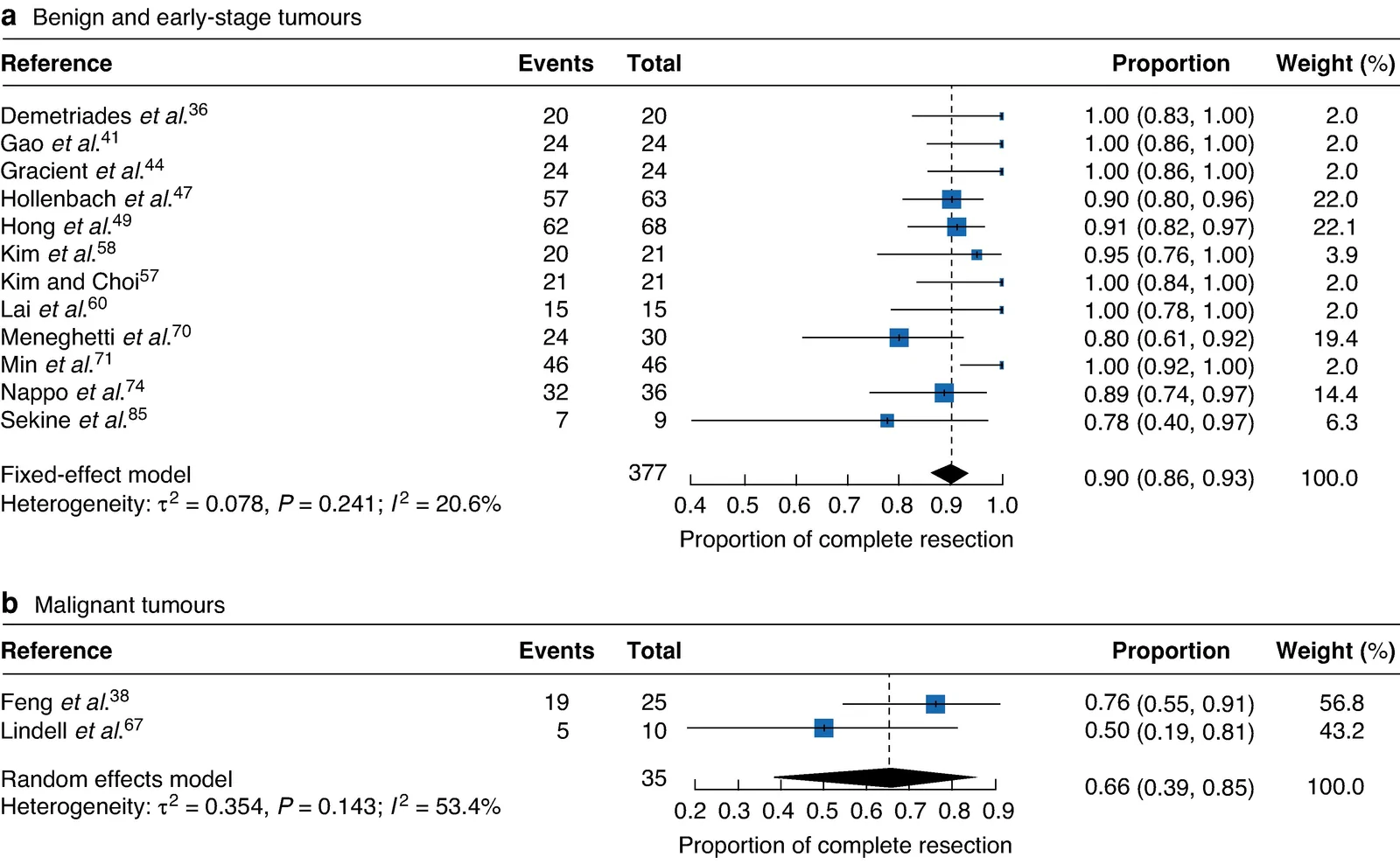
Forest plot reporting complete resection rate in transduodenal ampullectomy for benign and early-stage tumours and malignant tumours **a** Benign and early-stage tumours, **b** malignant tumours.

A total of 14 of 19 included studies provided data on complete resection in TDA, with 12 studies focusing on benign and early-stage tumours, and 2 on malignant tumours. The pooled rate of complete resection for benign and early-stage tumours was 90% (95% confidence interval (c.i.) 86% to 93%) with moderate heterogeneity (*I*^2^ = 20.6%; *P* = 0.241) (*Fig. 3a*). For malignant tumours, the pooled complete resection rate was 66% (95% c.i. 39% to 85%) (*Fig. 3b*) with moderate heterogeneity (*I*^2^ = 53.4%; *P* = 0.143).

**Fig. 4:**
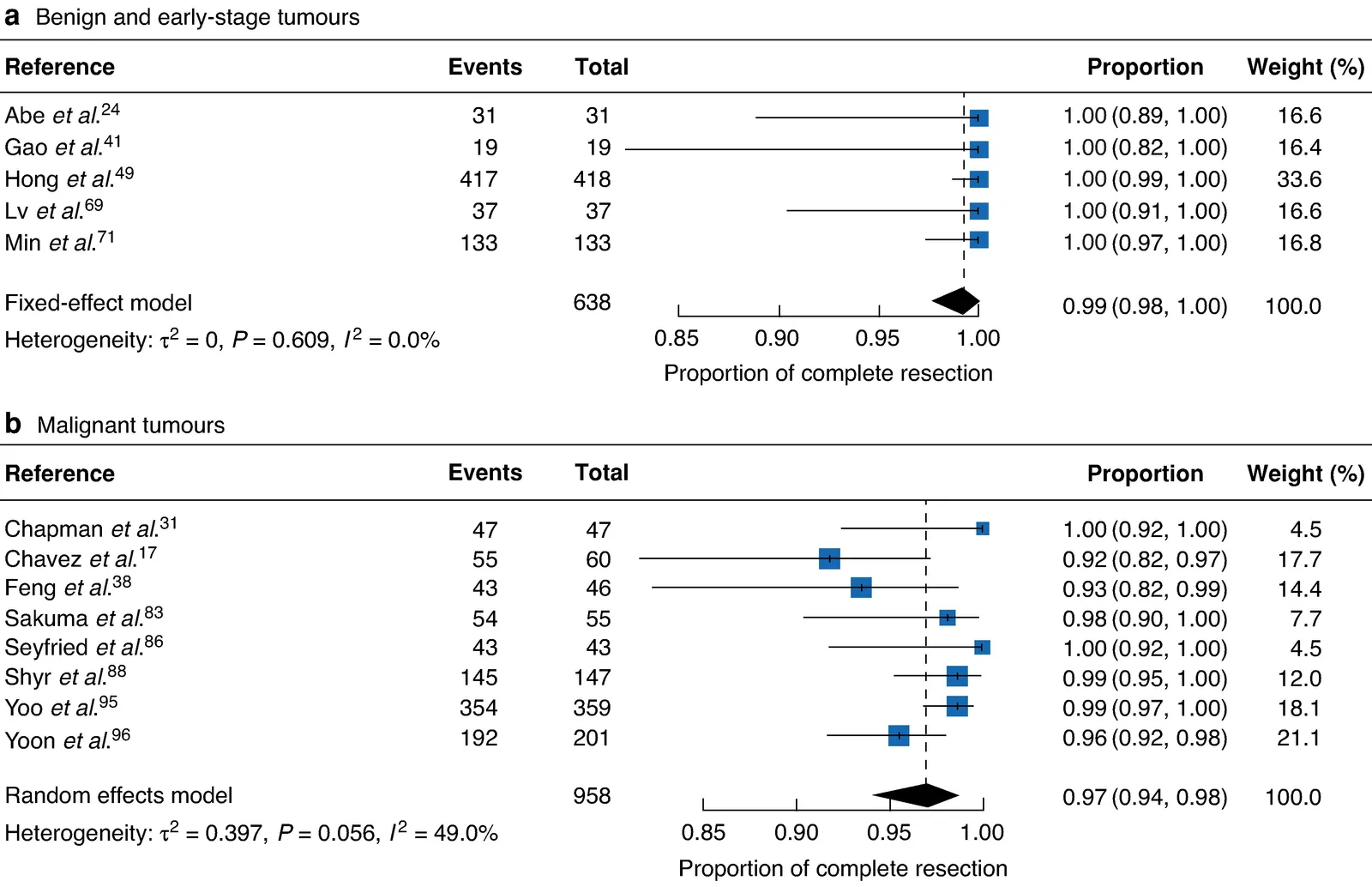
Forest plot reporting complete resection rate in pancreatoduodenectomy for benign and early-stage tumours and malignant tumours **a** Benign and early-stage tumours, **b** malignant tumours.

Data on complete resection in PD were presented in 16 of 19 included studies. The proportional meta-analysis for complete resection of benign and early-stage tumours, which included five studies, showed a pooled complete resection rate of 99% (95% c.i. 98% to 100%) with no heterogeneity (*I*^2^ = 0%; *P* = 0.609) (*Fig. 4a*). For malignant tumours, which included 11 studies, heterogeneity was high (*I*^2^ = 85.8%; *P* < 0.001); therefore, a sensitivity analysis was performed by excluding outliers. This sensitivity analysis showed a pooled complete resection rate of 97% (95% c.i. 94% to 98%) with moderate heterogeneity (*I*^2^ = 49.0%; *P* = 0.056) (*Fig. 4b*).

### Recurrence

Data on recurrence in ER were reported in 55 of 59 included studies. Proportion meta-analysis was performed, resulting in high heterogeneity (*I*^2^ = 70.8%; *P* < 0.001). After excluding outliers during sensitivity analysis, an acceptable heterogeneity (*I*^2^ = 50.5%; *P* < 0.001) was achieved (*Fig. S1*). The pooled recurrence rate for ER was found to be 12% (95% c.i. 11% to 14%).

Data on recurrence in TDA were available in 18 of 19 included studies. The proportional meta-analysis for recurrence of benign and early-stage tumours, which included 16 studies, showed a pooled recurrence rate of 12% (95% c.i. 9% to 16%) with low heterogeneity (*I*^2^ = 6.4%; *P* = 0.381) (*Fig. S2a*). For malignant tumours, two studies were included, but heterogeneity was high (*I*^2^ = 63.5%; *P* = 0.098), therefore, no pooled analysis was performed. The recurrence rate ranged from 48% to 80% (*Fig. S2b*).

A total of 16 of 19 included studies reported data on recurrence in PD. The proportional meta-analysis for recurrence in benign and early-stage tumours, which included seven studies, showed a pooled recurrence rate of 6% (95% c.i. 4% to 7%), with no heterogeneity (*I*^2^ = 0%; *P* = 0.589) (*Fig. S3a*). For malignant tumours, which included nine studies, there was substantial heterogeneity (*I*^2^ = 87.0%; *P* < 0.001). After excluding outliers in the sensitivity analysis, an acceptable heterogeneity (*I*^2^ = 56.1%; *P* = 0.034) was achieved, with a pooled recurrence rate of 16% (95% c.i. 12% to 21%) (*Fig. S3b*).

### Complications

Data on complications for ER were provided in 54 of 59 included studies. Proportional meta-analysis was attempted but revealed high heterogeneity (*I*^2^ = 84.3%; *P* < 0.001). Despite performing a sensitivity analysis, the heterogeneity remained high; therefore, no pooled analysis was performed. Complication rates for ER varied between 9% and 95% (*Fig. S4*).

Data on complications in TDA were reported in 17 of 19 included studies. Proportional meta-analysis showed a pooled complication rate of 32% (95% c.i. 28% to 37%) with moderate heterogeneity (*I*^2^ = 49%; *P* = 0.012) (*Fig. S5*).

Complications in PD were discussed in 17 of 19 included studies. High heterogeneity was observed (*I*^2^ = 91.9%; *P* < 0.001) despite performing sensitivity analysis. Hence, no pooled analysis was performed. Complication rates for PD ranged from 27% to 98% (*Fig. S6*).

### Publication bias

Funnel plots were used to assess publication bias for complete resection, recurrence, and complications across ER, TDA, and PD. Significant asymmetry was observed for ER (Egger’s test *P* = 0.006), TDA (*P* = 0.020), and PD (*P* = 0.003) for complete resection, indicating publication bias (*Fig. S7a-c*). For recurrence, significant asymmetry was seen in ER (*P* = 0.001) and TDA (*P* = 0.042), whereas no bias was detected for PD (*P* = 0.093) (*Fig. S8a–c*). For complications, the funnel plot was symmetrical for ER (*P* = 0.448) and PD (*P* = 0.055), suggesting no bias. For TDA, asymmetry was found (*P* = 0.013), indicating potential publication bias (*Fig. S9a–c*).

### Quality assessment

In general, the studies exhibited a moderate-to-serious risk of bias based on the ROBINS-I V2 assessment. The quality of the included studies was heterogeneous. Among the 80 studies, 42 (53%) were classified as having a moderate risk of bias, 35 (44%) were identified as having a serious risk of bias, and 3 (4%) had a critical risk of bias. The assessment of the risk of bias is summarized in *Table S1*.

## Discussion

This meta-analysis evaluated the outcomes of ER, TDA, and PD for ampullary tumours. Considerable heterogeneity was observed across studies, mainly due to variations in outcome definitions and preoperative diagnostic assessment, complicating direct comparisons. For benign tumours, ER seems a viable, less invasive option, despite slightly lower complete resection and higher recurrence rates compared with surgical resection. For malignant tumours, PD remains the only oncologically adequate option, as TDA achieves lower complete resection rates, has higher recurrence, and does not involve lymph node dissection. This heterogeneity limits firm recommendations, particularly for advanced adenomas and small ampullary cancers.

Accurate preoperative diagnosis is crucial, as distinguishing benign from malignant tumours is challenging, and misclassification can lead to undertreatment or overtreatment^98^. Despite advances in imaging, no consensus exists on the optimal preoperative diagnostic approach, and accuracy remains suboptimal^1,99,100^. Endoscopic ultrasound and preoperative biopsy demonstrate moderate accuracy (63% to 89% and 70% to 80%, respectively), reflecting a considerable risk of false-negative results^98,100–102^. In 32 ER studies of presumed benign tumours, 2–40% (median 10%) of adenomas were adenocarcinomas on final pathology, highlighting the risk of undertreatment. This variability underscores the difficulty of accurate preoperative diagnosis and the need for improved diagnostic methods.

Within benign ampullary tumours, a distinction can be made between incidentally discovered adenomas, where routine resection remains debated, and symptomatic adenomas with low-grade dysplasia, which can be removed by ER, with recurrence managed by surveillance, ablation, or re-endoscopy^82,103^. Adenomas with high-grade dysplasia or intraductal involvement are considered potentially premalignant lesions, for which complete resection is crucial^4,104^. ER may be suitable for high-risk adenomas if radical resection is technically feasible, although current studies do not distinguish these subgroups. For early-stage tumours (Tis and T1), the optimal therapeutic approach remains unclear. Several observational studies suggest that ER may be oncologically safe for T1 cancers without nodal involvement, but incomplete resection rates range from 9% to 43%^8,9,27^. Other studies^10,11,62^ report lymph node metastasis in 25%, indicating a substantial risk of undertreatment with ER and TDA. For malignant tumours, TDA is inappropriate, as it achieves lower complete resection rates and higher recurrence compared with PD. Although lymph node sampling has been proposed for TDA, available evidence shows that lymph node dissection during TDA is limited, non-standardized, and does not improve disease-free or overall survival^49,62^. Consequently, PD provides superior oncological outcomes for ampullary adenocarcinomas, consistent with previous reviews, meta-analyses and current guidelines^1,105,106^. Although minimally invasive PD was outside the scope of this review, a few studies^95,107^ suggest comparable oncological outcomes to open surgery.

Complications should also be considered when choosing the appropriate treatment. ER carries a lower complication risk than PD, making it a viable option for benign tumours. PD, associated with higher short- and long-term morbidity, should be reserved for advanced benign tumours or malignant tumours, where complete resection outweighs the risks. TDA has moderate morbidity, but limited oncological adequacy. Treatment selection requires careful evaluation of patient and tumour characteristics, diagnostic accuracy, and clinician experience.

This review has several limitations. First, high heterogeneity precluded meta-analysis for all outcomes. Most studies were retrospective, no RCTs were included, and the risk of bias was frequently moderate to high. Second, direct comparison of outcomes between ER, TDA, and PD was limited by heterogeneous definitions. In surgical cohorts, complete resection is defined histopathologically by negative resection margins (R0), whereas, in ER, margin assessment is often unreliable due to fragmentation of the specimen, and therefore complete resection is defined as the absence of macroscopically visible residual tumours at the first follow-up endoscopy. These definitions are not oncologically equivalent. Moreover, although complete resection and local recurrence are important indicators of treatment success, they do not necessarily translate into comparable overall or disease-free survival, particularly in T1 tumours where lymph node metastases occur in a substantial proportion of patients. In addition, the definitions and grading of postoperative complications differed both between and within treatment modalities. Surgical studies^108,109^ generally report complications using the Clavien–Dindo classification and often report only grade ≥ 3 complications. In contrast, ER studies report all complications without a standardized severity classification and apply varying thresholds. Third, some studies included patients with familial adenomatous polyposis who have a higher risk of recurrence, and most studies did not stratify between benign and early-stage tumours, precluding subgroup comparisons. Finally, temporal trends could not be assessed, as results were aggregated over long inclusion periods without stratification by year, learning curve, or centre volume.

In contrast to earlier reviews, this meta-analysis is the first to analyse comprehensively outcomes of ER, TDA, and PD for benign, early-stage, and malignant ampullary tumours. Despite the heterogeneity, it provides valuable insights into the outcomes of these treatment approaches. Given the limited high-quality prospective data, further prospective studies and well-conducted RCTs are needed to establish definitive guidelines. Future research should prioritize improving diagnostic accuracy to enable homogeneous patient groups, followed by comparative studies evaluating treatment strategies for benign and malignant tumours.

In conclusion, considerable heterogeneity exists in the management of ampullary tumours, and improvement in preoperative diagnostics is essential to optimize treatment selection. ER is the preferred treatment for benign tumours due to its low morbidity, despite a slightly increased recurrence rate. For early-stage tumours, no definitive conclusions can be drawn based on this review, though PD appears preferable. For malignant tumours, PD remains the primary treatment. Prospective studies are needed to optimize diagnostic and therapeutic strategies.

## Supplementary Material

zrag115_Supplementary_Data

## Data Availability

Data are available from the corresponding author on reasonable request. All data included in this meta-analysis are extracted from published studies, which are cited throughout the manuscript.
