# Supplementary material for: Therapeutic strategies and outcomes in the management of ampullary tumours: meta-analysis
Source: BJS Open. 2026 Aug 26;10(4):zrag115. doi: 10.1093/bjsopen/zrag115 (PMC13513765; doi:10.1093/bjsopen/zrag115)
Supplement: Supplementary file 1 [file zrag115_supplementary_data.zip › PRISMA_Flowchart_de Wilde.docx]

**Identification of studies via databases and registers**

Records identified through database searching n= 2633

PubMed n= 1437

Embase n= 1094

CINAHL n= 59

Cochrane n= 43

**Identification**

Duplicate records removed before screening n = 510

Records screened by abstract

n = 2123

Articles excluded based on abstract n =1855

**Screening**

Full text articles screened for eligibility

n = 268

Full-text articles excluded n = 188

- No full text available n = 39
- Only reference abstracts n = 32
- Less than 10 cases n = 18
- No subgroup analysis for ampullary tumors n = 23
- None of the outcome’s n = 44
- Wrong study design n =12
- Other n = 20

Studies included in review for analysis

n = 80

**Included**

Source: Page MJ, et al. BMJ 2021;372:n71. doi: 10.1136/bmj.n71.

This work is licensed under CC BY 4.0. To view a copy of this license, visit <https://creativecommons.org/licenses/by/4.0/>
