# Supplementary material for: Therapeutic strategies and outcomes in the management of ampullary tumours: meta-analysis
Source: BJS Open. 2026 Aug 26;10(4):zrag115. doi: 10.1093/bjsopen/zrag115 (PMC13513765; doi:10.1093/bjsopen/zrag115)
Supplement: Supplementary file 1 [file zrag115_supplementary_data.zip › Supplementary_Material.docx]

**Therapeutic strategies and outcomes in the management of Ampullary tumours: A meta-analysis**

Anouk J. de Wilde^1,2^, Jan Faes^2^, Karlijn E.P.E. Hermans^3^, Marc G. Besselink^4,5^, Jeska A. Fritzsche^5,6^ Matthias van Haele^7^, Jan-Werner Poley^8^, Rogier P. Voermans^5,6^, Judith De Vos-Geelen^3^, Steven W.M. Olde Damink^1,2,10^, Stefan A.W. Bouwense^1,2^

**Affiliations**

1: Department of Surgery and School of Nutrition and Translational Research in Metabolism (NUTRIM), Maastricht University, Maastricht, the Netherlands

2: Department of Surgery, Maastricht University Medical Centre+, Maastricht, the Netherlands

3: Department of Internal Medicine, Division of Medical Oncology, GROW – Research Institute for Oncology & Reproduction, Maastricht University Medical Centre+, Maastricht, the Netherlands

4: Amsterdam UMC, location University of Amsterdam, Department of Surgery, Amsterdam, the Netherlands.

5: Cancer Centre Amsterdam, Amsterdam, the Netherlands.

6: Department of Gastroenterology and Hepatology, Amsterdam Gastroenterology Endocrinology Metabolism, Amsterdam UMC, University of Amsterdam, Amsterdam, the Netherlands.

7: Department of Pathology, Maastricht University Medical Centre+, Maastricht, the Netherlands

8: Department of Gastroenterology, Maastricht University Medical Centre+, Maastricht, the Netherlands.

9: Department of General, Visceral, Vascular and Transplantation Surgery, University Hospital Essen, Essen Germany

**Corresponding author after publication:**

Stefan A.W. Bouwense

Tel: +31 630530879; E-mail: [stefan.bouwense@mumc.nl](mailto:stefan.bouwense@mumc.nl)

**Corresponding author during submission:**

Anouk J. de Wilde

Tel: +31 650906078; E-mail: [anouk.de.wilde@mumc.nl](mailto:anouk.de.wilde@mumc.nl)

**Supplementary Materials - Index**

| **Supplementary Appendixes** |  |
| --- | --- |
| A. Search PubMed B. Search Embase C. Search Cochrane Library D. Search CINAHL | *pag. 3*  *pag. 4*  *pag. 5-6*  *pag. 7* |
| **Supplementary Tables** |  |
| Table 1. Quality analysis of included studies | *pag. 8-9* |
| **Supplementary Figures** |  |
| Figure 1. Forest plot reporting recurrence rate in ER  Figure 2. Forest plot reporting recurrence in TDA  Figure 3. Forest plot reporting recurrence in PD  Figure 4. Forest plot reporting complications in ER  Figure 5. Forest plot reporting complications in TDA  Figure 6. Forest plot reporting complications in PD | *pag. 10*  *pag. 11*  *pag. 12*  *pag. 13*  *pag. 14*  *pag. 15* |
| Figure 7 a-c. Funnel plots complete resection  Figure 8 a-c. Funnel plots recurrence  Figure 9 a-c. Funnel plots complications | *pag. 16*  *pag. 17*  *pag. 18* |

**Supplementary Appendixes**

A. **Search PubMed**

((("Ampulla of Vater"[Mesh]) OR (Vater ampulla) OR (Ampulla of vater)) OR ((Ampullary) OR (Hepatopancreatic ampulla) OR (Vater* ampulla)) OR ((Major duodenal papilla) OR (Greater duodenal papilla))) AND ((("Neoplasms"[Mesh]) OR ("Common Bile Duct Neoplasms"[Mesh]) OR (Neoplas*)) OR ((Tumor*) OR (Tumour*)) OR (Cancer*) OR (Malignan*) OR (Carcinom*)) AND ((("Pancreaticoduodenectomy"[Mesh]) OR (Whipple) OR (Pylorus Preserving Pancreaticoduodenectomy) OR (Pylorus Resecting Pancreaticoduodenectomy)) OR ((Ampullectomy) OR (Papillectomy) OR (Transduodenal *ectomy)) OR (Endoscopic resection) OR (local resection)) AND ((complete resection) OR (successful resection) OR (primary success) OR (complication*) OR (recurrence))

B. **Search Embase**

| **#** | **Query** | **Results** |
| --- | --- | --- |
| 1 | exp Vater papilla carcinoma/ or exp "ampulla of Vater"/ or exp Vater papilla/ | 5,733 |
| 2 | Ampulla of Vater.mp. | 5,300 |
| 3 | Ampullary.mp. | 7,209 |
| 4 | Vater* ampulla.mp. | 287 |
| 5 | exp neoplasm/ | 5,536,836 |
| 6 | Neoplas*.mp. | 1,137,650 |
| 7 | exp adenocarcinoma/ | 292,309 |
| 8 | Tumor*.mp. | 3,648,065 |
| 9 | Tumour*.mp. | 437,361 |
| 10 | Cancer*.mp. | 4,554,998 |
| 11 | Malignan*.mp. | 1,137,428 |
| 12 | Carcinom*.mp. | 1,417,612 |
| 13 | 1 or 2 or 3 or 4 | 12,919 |
| 14 | 5 or 6 or 7 or 8 or 9 or 10 or 11 or 12 | 7,205,409 |
| 15 | exp pancreaticoduodenectomy/ | 27,890 |
| 16 | Whipple.mp. | 4,953 |
| 17 | Pylorus preserving pancreaticoduodenectomy.mp. | 946 |
| 18 | Pylorus resecting pancreaticoduodenectomy.mp. | 22 |
| 19 | exp ampullectomy/ | 110 |
| 20 | Papillectomy.mp. | 650 |
| 21 | exp endoscopic surgery/ | 338,390 |
| 22 | Transduodenal ampullectomy.mp. | 102 |
| 23 | Local resection.mp. | 2,567 |
| 24 | 15 or 16 or 17 or 18 or 19 or 20 or 21  or 22 or 23 | 367,877 |
| 25 | 13 and 14 | 9,627 |
| 26 | 24 and 25 | 4,374 |
| 27 | Complete resection rate.mp. | 1,167 |
| 28 | Succesful resection.mp. | 4 |
| 29 | Primary success.mp. | 1,477 |
| 30 | Radicality.mp. | 2,902 |
| 31 | Recurrence.mp. | 767,695 |
| 32 | exp complication/su [Surgery] | 38,211 |
| 33 | 27 or 28 or 29 or 30 or 31 or 32 | 807,277 |
| 34 | 26 and 33 | 1,094 |

C. **Search Cochrane Library**

ID	Search Hits

#1	MeSH descriptor: [Ampulla of Vater] explode all trees and with qualifier(s): [abnormalities - AB, surgery - SU]	61

#2	Major duodenal papilla	71

#3	Ampullary	230

#4	Ampulla of Vater	295

#5	#1 or #2 or #3 or #4	538

#6	MeSH descriptor: [Duodenal Neoplasms] explode all trees and with qualifier(s): [surgery - SU] 18

#7	Ampullary neoplasm 35

#8	Neoplasms	92163

#9	MeSH descriptor: [Neoplasms] explode all trees and with qualifier(s): [surgery - SU] 17171

#10	Cancer 219713

#11	Tumor 79798

#12	Tumour	79767

#13	Malignancy	8641

#14	#6 or #7 or #8 or #9 or #10 or #11 or #12 or #13	258039

#15	#5 and #14	343

#16	MeSH descriptor: [Pancreaticoduodenectomy] explode all trees	444

#17	Whipple	310

#18	MeSH descriptor: [Endoscopy, Gastrointestinal] explode all trees 6215 #19	Endoscopic resection 2866

#20	Endoscopic surgery	10482

#21	pylorus preserving pancreaticoduodenectomy	155

#22	#16 or #17 or #18 or #19 or #20 or #21	17058

#23	#15 and #22	134

#24	Complete resection rate	2723

#25	Primary success	16893

#26	MeSH descriptor: [Recurrence] explode all trees	14536 #27	Radicality	135

#28	MeSH descriptor: [Postoperative Complications] explode all trees 49407 #29	#24 or #25 or #26 or #27 or #28	81592

#30	#23 and # 29 43

D. **Search CINAHL**

| S35 | AB S6 AND S14 AND S25 AND S32 | Expanders - Apply equivalent subjects Search modes - Boolean/Phrase | 59 |
| --- | --- | --- | --- |
| S34 | AB S6 | Expanders - Apply equivalent subjects Search modes - Boolean/Phrase | 587 |
| S33 | S26 AND S32 | Expanders - Apply equivalent subjects Search modes - Boolean/Phrase | 1,896 |
| S32 | S27 OR S28 OR S29 OR S30 OR S31 | Expanders - Apply equivalent subjects Search modes - Boolean/Phrase | 139,619 |
| S31 | complications surgery | Expanders - Apply equivalent subjects Search modes - Boolean/Phrase | 19,260 |
| S30 | Recurrence | Expanders - Apply equivalent subjects Search modes - Boolean/Phrase | 121,489 |
| S29 | Radicality | Expanders - Apply equivalent subjects Search modes - Boolean/Phrase | 147 |
| S28 | primary succession | Expanders - Apply equivalent subjects Search modes - Boolean/Phrase | 5 |
| S27 | Complete resection rate | Expanders - Apply equivalent subjects Search modes - Boolean/Phrase | 454 |
| S26 | S15 OR S25 | Expanders - Apply equivalent subjects Search modes - Boolean/Phrase | 7,869 |
|  | S16 OR S17 OR S18 OR S19 OR S20 OR S21 OR S22 OR | Expanders - Apply equivalent subjects |  |
| S25 | S23 OR S24 | Search modes - Boolean/Phrase | 7,472 |
| S24 | Local resection | Expanders - Apply equivalent subjects Search modes - Boolean/Phrase | 1,138 |
| S23 | endoscopic resection | Expanders - Apply equivalent subjects Search modes - Boolean/Phrase | 3,451 |
| S22 | Transduodenal ampullectomy | Expanders - Apply equivalent subjects Search modes - Boolean/Phrase | 11 |
| S21 | Papillectomy | Expanders - Apply equivalent subjects Search modes - Boolean/Phrase | 87 |
| S20 | Ampullectomy | Expanders - Apply equivalent subjects Search modes - Boolean/Phrase | 68 |
| S19 | Pylorus resecting pancreaticoduodenectomy | Expanders - Apply equivalent subjects Search modes - Boolean/Phrase | 2 |

**Supplementary Tables**

*Supplementary Table 1.* Quality analysis of included studies

| Study | D1 | D2 | D3 | D4 | D5 | D6 | D7 | Overall |
| --- | --- | --- | --- | --- | --- | --- | --- | --- |
| Abe et al |  |  |  |  |  |  |  |  |
| Ahn et al |  |  |  |  |  |  |  |  |
| Alali et al |  |  |  |  |  |  |  |  |
| Alvarez et al |  |  |  |  |  |  |  |  |
| Attila et al |  |  |  |  |  |  |  |  |
| Bohnacker et al |  |  |  |  |  |  |  |  |
| Boix et al |  |  |  |  |  |  |  |  |
| Catalano et al |  |  |  |  |  |  |  |  |
| Chapman et al |  |  |  |  |  |  |  |  |
| Chavez et al |  |  |  |  |  |  |  |  |
| Cheng et al |  |  |  |  |  |  |  |  |
| Choi et al, 2024 |  |  |  |  |  |  |  |  |
| Choi et al, 2022 |  |  |  |  |  |  |  |  |
| de Palma et al |  |  |  |  |  |  |  |  |
| Dembinski et al |  |  |  |  |  |  |  |  |
| Demetriades et al |  |  |  |  |  |  |  |  |
| Dubois et al |  |  |  |  |  |  |  |  |
| Feng et al |  |  |  |  |  |  |  |  |
| Fritzsche et al |  |  |  |  |  |  |  |  |
| Gambitta et al |  |  |  |  |  |  |  |  |
| Gao et al |  |  |  |  |  |  |  |  |
| Ghidrim et al |  |  |  |  |  |  |  |  |
| Gondran et al |  |  |  |  |  |  |  |  |
| Gracient et al |  |  |  |  |  |  |  |  |
| Grobmyer et al |  |  |  |  |  |  |  |  |
| Harano et al |  |  |  |  |  |  |  |  |
| Hollenbach et al |  |  |  |  |  |  |  |  |
| Hong et al, 2018 |  |  |  |  |  |  |  |  |
| Hong et al, 2021 |  |  |  |  |  |  |  |  |
| Hwang et al |  |  |  |  |  |  |  |  |
| Irani et al |  |  |  |  |  |  |  |  |
| Jeanniard et al |  |  |  |  |  |  |  |  |
| Jung et al |  |  |  |  |  |  |  |  |
| Jung et al |  |  |  |  |  |  |  |  |
| Kang et al |  |  |  |  |  |  |  |  |
| Kawashima et al |  |  |  |  |  |  |  |  |
| Kim et al, 2017 |  |  |  |  |  |  |  |  |
| Kim et al, 2011 |  |  |  |  |  |  |  |  |
| Klein et al |  |  |  |  |  |  |  |  |
| Lai et al |  |  |  |  |  |  |  |  |
| Laleman et al |  |  |  |  |  |  |  |  |
| Lee et al, 2024 (Junghwan) |  |  |  |  |  |  |  |  |
| Lee et al, 2024 (Jonghyun) |  |  |  |  |  |  |  |  |
| Lee et al, 2017 |  |  |  |  |  |  |  |  |
| Lee et al, 2016 |  |  |  |  |  |  |  |  |
| Li et al |  |  |  |  |  |  |  |  |
| Lindell et al |  |  |  |  |  |  |  |  |
| Lu Shunli et al |  |  |  |  |  |  |  |  |
| Lv et al |  |  |  |  |  |  |  |  |
| Meneghetti et al |  |  |  |  |  |  |  |  |
| Min et al |  |  |  |  |  |  |  |  |
| Miyamoto et al |  |  |  |  |  |  |  |  |
| Muro et al |  |  |  |  |  |  |  |  |
| Napoleon et al |  |  |  |  |  |  |  |  |
| Nappo et al |  |  |  |  |  |  |  |  |
| Norton et al |  |  |  |  |  |  |  |  |
| Odemis et al |  |  |  |  |  |  |  |  |
| Onkendi et al |  |  |  |  |  |  |  |  |
| Patel et al |  |  |  |  |  |  |  |  |
| Perez et al |  |  |  |  |  |  |  |  |
| Ridtitid et al |  |  |  |  |  |  |  |  |
| Russel et al |  |  |  |  |  |  |  |  |
| Sahar et al |  |  |  |  |  |  |  |  |
| Sakai et al |  |  |  |  |  |  |  |  |
| Sakuma et al |  |  |  |  |  |  |  |  |
| Saurin et al |  |  |  |  |  |  |  |  |
| Sekine et al |  |  |  |  |  |  |  |  |
| Seyfried et al |  |  |  |  |  |  |  |  |
| Shim et al |  |  |  |  |  |  |  |  |
| Shyr et al |  |  |  |  |  |  |  |  |
| Suzuki et al |  |  |  |  |  |  |  |  |
| Tringali et al |  |  |  |  |  |  |  |  |
| van der Wiel et al |  |  |  |  |  |  |  |  |
| Wang et al |  |  |  |  |  |  |  |  |
| Will et al |  |  |  |  |  |  |  |  |
| Xie et al |  |  |  |  |  |  |  |  |
| Yamamoto et al |  |  |  |  |  |  |  |  |
| Yoo et al |  |  |  |  |  |  |  |  |
| Yoon et al |  |  |  |  |  |  |  |  |
| Zadorova et al |  |  |  |  |  |  |  |  |
|  |  |  |  |  |  |  |  |  |
| Legend |  | Low |  | Moderate |  | Serious |  | Critical |

D1; confounding, D2; participant selection, D3; classification of interventions, D4; deviations from intended interventions, D5; missing data, D6; outcome measurement, D7; selection of the reported outcome.

**Supplementary Figures**

*Supplementary Figure 1.* Forest plot reporting recurrence rate in endoscopic resection for benign and early-stage tumours.

*Supplementary Figure 2.* Forest plot reporting recurrence rate in transduodenal ampullectomy for (a) benign and early-stage tumours and for (b) malignant tumours.

*Supplementary Figure 3.* Forest plot reporting recurrence rate in pancreatoduodenectomy for (a) benign and early-stage tumours and for (b) malignant tumours.

*Supplementary Figure 4.* Forest plot reporting complications in endoscopic resection for benign and early-stage tumours.

*Supplementary Figure 5.* Forest plot reporting complications in transduodenal ampullectomy.

*Supplementary Figure 6.* Forest plot reporting complications in pancreatoduodenectomy.

*Supplementary Figure 7a:* Funnel plots analysing publication bias for complete resection in endoscopic resection.
*Supplementary Figure 7b:* Funnel plots analysing publication bias for complete resection in transduodenal ampullectomy.
*Supplementary Figure 7c:* Funnel plots analysing publication bias for complete resection in pancreatoduodenectomy.

*Supplementary Figure 8a:* Funnel plots analysing publication bias for recurrence in endoscopic resection.

*Supplementary Figure 8b:* Funnel plots analysing publication bias for recurrence in transduodenal ampullectomy.
*Supplementary Figure 8c:* Funnel plots analysing publication bias for recurrence in pancreatoduodenectomy.

*Supplementary Figure 9a:* Funnel plots analysing publication bias for complications in endoscopic resection.
*Supplementary Figure 9b:* Funnel plots analysing publication bias for complications in transduodenal ampullectomy.

*Supplementary Figure 9c:* Funnel plots analysing publication bias for complications in pancreatoduodenectomy.
